# Research into the biological differences and targets in lung cancer patients with diverse immunotherapy responses

**DOI:** 10.3389/fimmu.2022.1014333

**Published:** 2022-09-16

**Authors:** Xunlang Zhang, Xinhui Wu, Huang Huang, Kangming Du, Yingying Nie, Peiyuan Su, Yuefei Li

**Affiliations:** ^1^Department of Geriatric, Hospital of Chengdu University of Traditional Chinese Medicine, Chengdu, China; ^2^Department of Cardiology, Hospital of Chengdu University of Traditional Chinese Medicine, Chengdu, China; ^3^Department of Vascular Surgery, Hospital of Chengdu University of Traditional Chinese Medicine, Chengdu, China; ^4^Department of Ophthalmology, Ineye Hospital of Chengdu University of Traditional Chinese Medicine, Chengdu, China; ^5^Department of Cardiothoracic Surgery, Hospital of Chengdu University of Traditional Chinese Medicine, Chengdu, China; ^6^Department of Anesthesiology Operating Room, Hospital of Chengdu University of Traditional Chinese Medicine, Chengdu, China

**Keywords:** lung cancer, immunotherapy, gender, monocytes, molecules

## Abstract

**Background:**

Immunotherapy has gradually become an important therapy option for lung cancer patients.

**Methods:**

The Cancer Genome Atlas (TCGA) and Gene Expression Omnibus (GEO) databases were responsible for all the public data.

**Results:**

In our study, we firstly identified 22 characteristic genes of NSCLC immunotherapy response using the machine learning algorithm. Molecule subtyping was then conducted and two patient subtypes were identified Cluster1 and Cluster2. Results showed that Cluster1 patients had a lower TIDE score and were more sensitive to immunotherapy in both TCGA and combined GEO cohorts. Biological enrichment analysis showed that pathways of epithelial-mesenchymal transition (EMT), apical junction, KRAS signaling, myogenesis, G2M checkpoint, E2F targets, WNT/β-catenin signaling, hedgehog signaling, hypoxia were activated in Cluster2 patients. Genomic instability between Cluster1 and Cluster2 patients was not significantly different. Interestingly, we found that female patients were more adaptable to immunotherapy. Biological enrichment revealed that compared with female patients, pathways of MYC target, G2M checkpoints, mTORC1 signaling, MYC target, E2F target, KRAS signaling, oxidative phosphorylation, mitotic spindle and P53 pathway were activated. Meanwhile, monocytes might have a potential role in affecting NSCLC immunotherapy and underlying mechanism has been explored. Finally, we found that SEC14L3 and APCDD1L were the underlying targets affecting immunotherapy, as well as patients survival.

**Conclusions:**

These results can provide direction and guidance for future research focused on NSCLC immunotherapy.

## Introduction

With recent advances in biotechnology, researchers have gained a deeper understanding of tumor genomics and immunosuppressive tumor microenvironments, also leading to the change of treatment concepts for tumors (1). Nowadays, personalized precision therapy is gradually available for the treatment of tumors instead of tumor type-centered therapies (2). Annually, approximately 1.76 million people die from lung cancer, which is a serious threat to public health (3). Targeted therapies and immunotherapies based on EGFR, KRAS, and PD-L1 in individual patients have achieved promising results (4). Furthermore, researchers have classified tumor microenvironments (TME) as “immune inflammation”, “immune evasion”, and “immune desert” and adopted appropriate treatment methods according to these categories (5). Meanwhile, modern tumor treatment is gradually becoming more individualized.

For the moment, surgery, along with postoperative systemic therapy can still provide good therapeutic gain for resectable lung cancer patients (6). Nevertheless, insidious early symptoms usually lead to the challenge of early diagnosis and disease advancement has been occurred when most patients are first diagnosed (6). For advanced lung cancer, especially for those who lost surgery chance, therapy options are limited. The past decade has seen tremendous advancements in medical technology and basic biological research and therefore, cancer immunotherapy has gained public attention. The advent of immunotherapy has revolutionized lung cancer treatment and has become a vital biological therapy, among which immune checkpoint inhibitors (ICIs) indicated promising effects (7). Despite this, not all patients respond to immunotherapy well, indicating that immunotherapeutic response may vary according to the individual’s biological characteristics. An example, according to previous high-quality studies, tumor mutational burden (TMB) appears to be a promising immunotherapy biomarker. As of yet, there are no satisfactory markers for predicting lung cancer immune response. As a consequence, the identification of new and effective markers to assess lung cancer patients’ immunotherapy response is of great significance.

In our study, we comprehensively explored the underlying differences between immunotherapy responders and non-responders of non-small cell lung cancer (NSCLC). We identified characteristic genes based on machine learning and performed molecular subtyping to screen patients with different responses to immunotherapy. Two patient subtypes Cluster1 and Cluster2 were identified, among which Cluster1 patients were more adaptable to immunotherapy. Interestingly, we found that female patients were more adaptable to immunotherapy; monocytes have a potential role in affecting NSCLC immunotherapy; SEC14L3 and APCDD1L were the underlying targets affecting immunotherapy, as well as patients survival. These results can provide direction and guidance for future research focused on NSCLC immunotherapy

## Methods

### Assessment of data

Gene expression profiles and corresponding clinical parameters of NSCLC patients were downloaded from the public databases, The Cancer Genome Atlas (TCGA) and Gene Expression Omnibus (GEO). For TCGA, the gene expression profiles were obtained from the GDC interactive interface in a “STAR-Counts” file. Then, the gene expression of transcripts per kilobase million (TPM) form was extracted. For GEO, the GSE30219, GSE37745 and GSE50081 were identified and the platforms of which were all GPL570. The ‘affy’ and ‘simpleaffy’ R packages were utilized to contextualize and normalize the raw ‘CEL’ files of microarray sequencing. The batch effects of different datasets were eliminated based on the “Sva” package. The patients with complete gene expression profiles and corresponding clinical parameter were included in this study, otherwise, were excluded. The baseline information of enrolled patients were shown in **Tables S1–S4**.

### Immunotherapy response

Evaluation of patients’ responses to immunotherapy was realized through Tumor Immune Dysfunction and Exclusion (TIDE) website (8). The cancer type was selected as “NSCLC”. The “Previous immunotherapy” was set as “No”. Patients were assigned a TIDE score based on their normalized expression profile, of which TIDE scores > 0 were non-responders and < 0 were responders. The Submap module in the GenePattern website was used to quantify the response probability of a single sample or a subtype to immunotherapy (https://cloud.genepattern.org/gp).

### Machine learning and molecular subtyping

For the identification of the characteristic genes, LASSO logistic regression and support vector machine recursive feature elimination (SVM-RFE) algorithms were utilized (9). Machine learning algorithms were utilized to select the optimized variables through dimensionality reduction. A consensus clustering analysis was performed using the ConsensusClusterPlus package and the resamplings of which was 1,000.

### Biological enrichment and genomic analysis

The potential biological differences between specific groups were determined through Gene Set Enrichment Analysis (GSEA) and clueGO analysis (10). The reference gene set was the Hallmark, c2.cp.kegg.v7.5.1.symbols and c5.go.v7.5.1.symbols gene set. Somatic nonsynonymous mutations occurring per megabase in NSCLC samples were used to account for the tumor mutational burden (TMB). Copy number variation (CNV) burden was calculated using the GISTIC 2.0 and the input file was obtained from the https://gdac.broadinstitute.org/%20website, including segmented copy number profiles and genomic positions of amplified regions. The mRNAsi and EREG-mRNAsi score reflecting tumor stemness were get from the previous study (11).

### Immune microenvironment quantification

Quantification of infiltration of 22 immune cells was conducted with the CIBERSORT algorithm (12).

### Single cell analysis

The single-cell analysis was performed based on the TISCH website (http://tisch.comp-genomics.org/home/). Aside from providing detailed cell-type annotations, TISCH also allows for the exploration of TME across a variety of cancer types (13).

### Statistical analysis

All statistical analysis was conducted using R software v4.0.0. The Mann-Whitney U test was used for non-normally distributed variables. Statistical differences between continuous variables with normal distributions were determined by the Student-T test. Kaplan-Meier (KM) survival curves were utilized to determine the prognosis difference in different groups.

## Results

### Identification of characteristic genes

**Figure 1:**
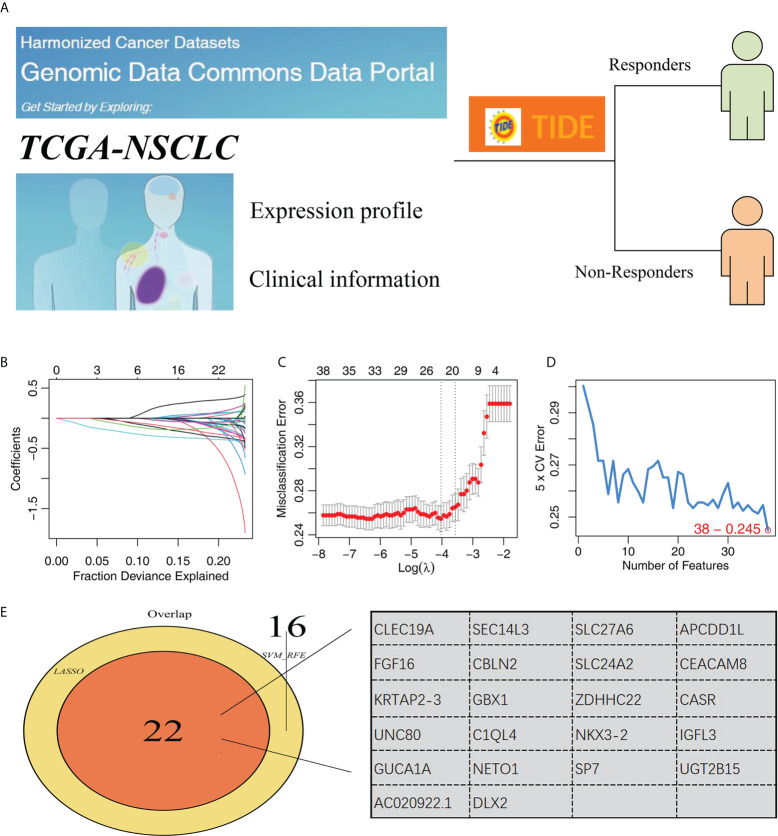
Identification of characteristic genes of NSCLC immunotherapy. **(A)** TIDE algorithm was performed to evaluate the immunotherapy of NSCLC patients, of which TIDE scores > 0 were non-responders and < 0 were responders; **(B, C)** LASSO logistic regression; **(D)** SVM-RFE algorithm; **(E)** Two algorithms identified 34 characteristic genes.

The whole chart of this study was shown in **Figure S1**. Firstly, through the TIDE analysis, we divided the NSCLC patients in TCGA cohort into two groups, immunotherapy responders and non-responders, according to the calculated TIDE score (**Figure 1A**). Subsequently, SVM-RFE algorithm and LASSO logistic regression were utilized to screen the optimal variable on immunotherapy response (**Figures 1B–D**). Ultimately, 22 genes were selected as the characteristic genes of NSCLC immunotherapy response, including CLEC19A, SEC14L3, SLC27A6, APCDD1L, FGF16, CBLN2, SLC24A2, CEACAM8, KRTAP2-3, GBX1, ZDHHC22, CASR, UNC80, C1QL4, NKX3-2, IGFL3, GUCA1A, NETO1, SP7, UGT2B15, AC020922.1 and DLX2 (**Figure 1E**).

### Genotyping of NSCLC patients

**Figure 2:**
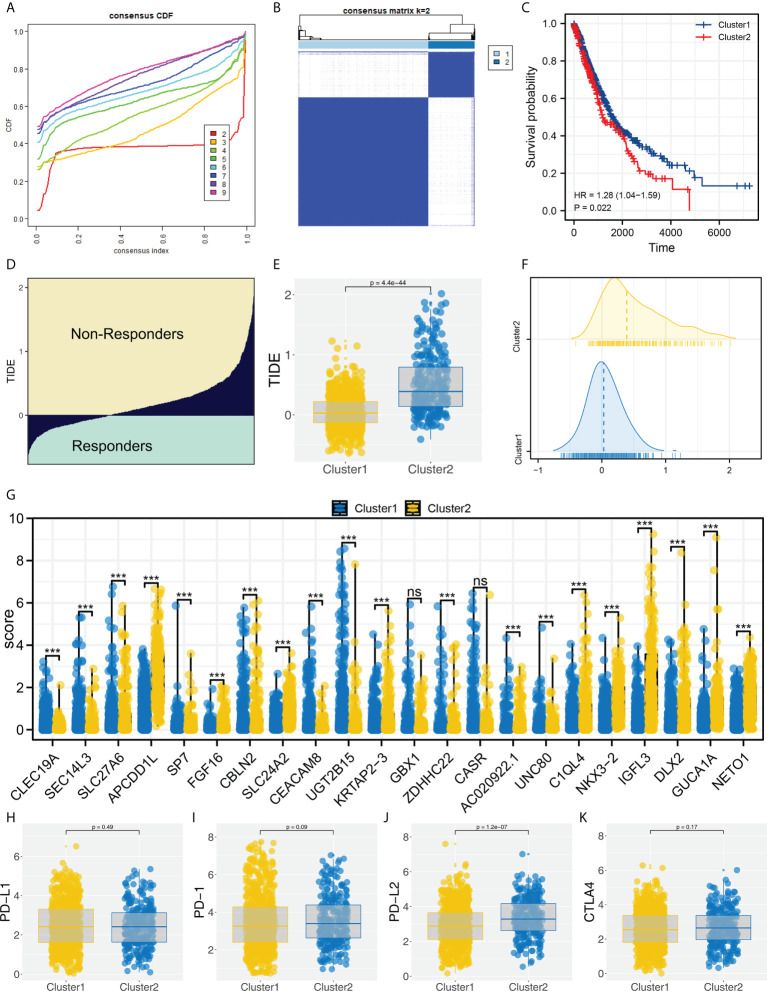
Molecular typing based on identified characteristic genes. **(A)** ConsensusClusterPlus package was used for molecular typing; **(B)** Two subtypes provide the best differentiation; **(C)** KM survival curve showed that Cluster2 patients had a worse prognosis; **(D)** The calculated TIDE score of TCGA patients, of which TIDE scores > 0 were non-responders and < 0 were responders; **(E, F)** The patients in Cluster2 had a higher TIDE score; **(G)** The expression level of characteristic genes in Cluster1 and Cluster2 patients, ns = P < 0.05, *** = P < 0.001; **(H–K)** The PD-1, PD-L1, PD-L2 and CTLA4 expression in Cluster1 and Cluster2 patients.

Based on the identified characteristic genes, we performed genotyping using the ConsensusClusterPlus R package (**Figure 2A**). We found two subtypes had the best discrimination (**Figure 2B** and **Figure S2**). KM survival indicated a worse overall survival (OS) in Cluster2 patients compared to Cluster1 patients (**Figure 2C**, HR = 1.28, P = 0.022). Meanwhile, the patients in Cluster2 had a higher TIDE score than Cluster1 patients (**Figures 2D–F**). The expression of all 22 of these characteristic genes differed between Cluster1 and Cluster2 (**Figure 2G**). Then, we assessed the CTLA4, PD-L2, PD-1 and PD-L1 expression in Cluster1 and Cluster2 patients (**Figures 2H–K**). Corresponding results showed that Cluster2 patients had a higher PD-L2 expression than Cluster1 patients (**Figure 2J**).

### Cluster1 patients are more sensitive to immunotherapy

**Figure 3:**
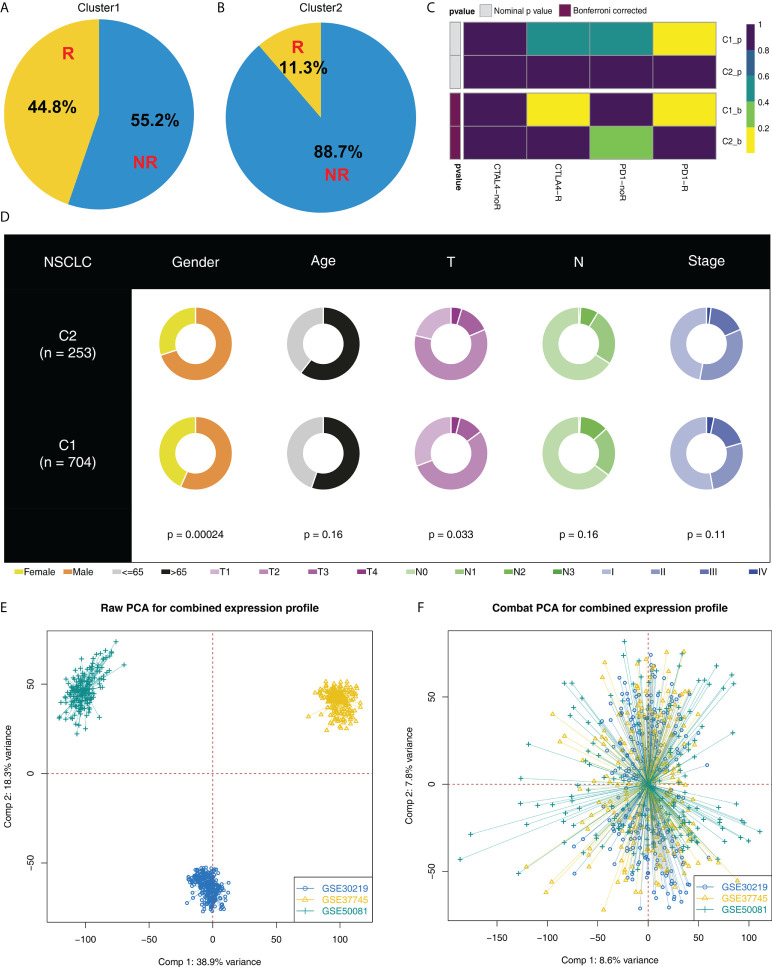
Cluster1 and Cluster2 had different immunotherapy response. **(A, B)** The proportion of immunotherapy responders in Cluster1 and Cluster2 patients; **(C)** Submap algorithm indicated that the Cluster1 patients are sensitive to both PD-1 and CTLA4 therapy; **(D)** Clinical features difference in Cluster1 and Cluster2 patients; **(E, F)** Sva package was used for data combination and batch effect reduction of GSE30219, GSE37745 and GSE50081.

Moreover, we found an increased number of immunotherapy responders in Cluster1 patients than in Cluster2 patients (**Figures 3A, B**, 44.8% *vs*. 11.3%). Furthermore, according to the result from submap analysis, there is an increased sensitivity to PD-1 and CTLA4 therapy among Cluster1 patients (**Figure 3C**). Clinical features analysis indicated that the Cluster2 patients were associated with more aggressive clinical parameters, as well as a high proportion of male patients (**Figure 3D**). Additionally, we attempt to validate our results in GEO cohorts. GSE30219, GSE37745 and GSE50081 were selected (**Figure 3E**). Sva package was utilized for data combination and batch effect reduction (**Figure 3F**).

### Validation in the combined GEO cohort

**Figure 4:**
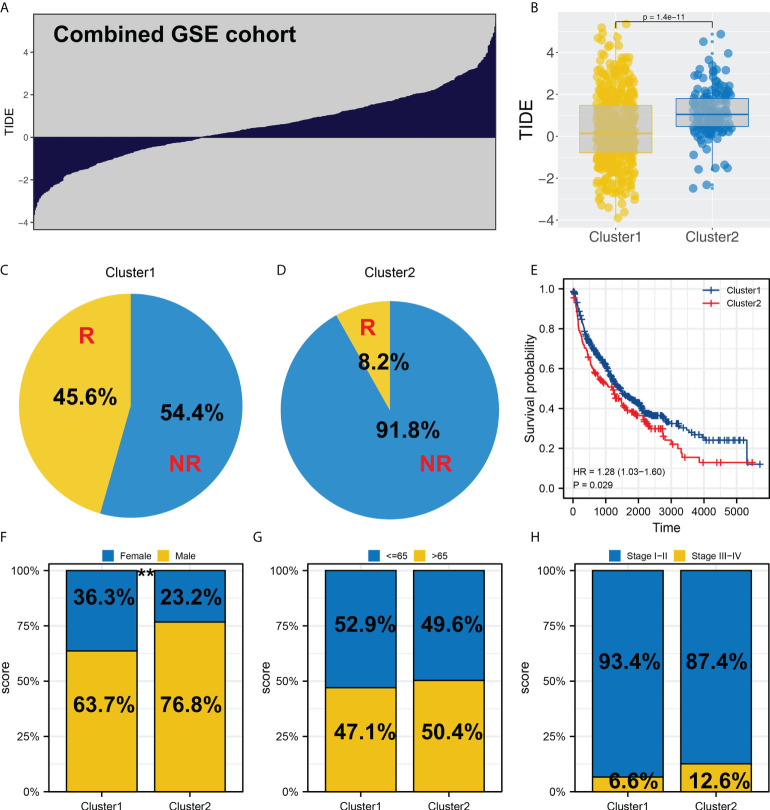
Validation in the GEO cohort. **(A)** TIDE analysis was performed in the combined GEO cohort; **(B)** Cluster2 had a higher TIDE score than Cluster1; **(C, D)** The proportion of immunotherapy responders in Cluster1 and Cluster2 patients; **(E)** KM survival curve of Cluster1 and Cluster2 patients in GEO cohort; **(F-H)** Clinical differences between Cluster1 and Cluster2, ** = P < 0.01.

In the combined GEO cohort, we also calculated the TIDE score (**Figure 4A**). Also, an increased TIDE score was observed among Cluster2 patients, indicating a lower percentage of immunotherapy responders (**Figures 4B–D**, 8.2% *vs*. 45.6%). Meanwhile, patients in Cluster2 had a poorer prognosis than those in Cluster1, consistent with the result of TCGA (**Figure 4E**). Interestingly, the result of the GSE cohort also indicated a higher percentage of female patients in Cluster1 (**Figure 4F**). However, no significant difference was found in age and stage parameters (**Figures 4G, H**).

### Biological and genomic features difference

**Figure 5:**
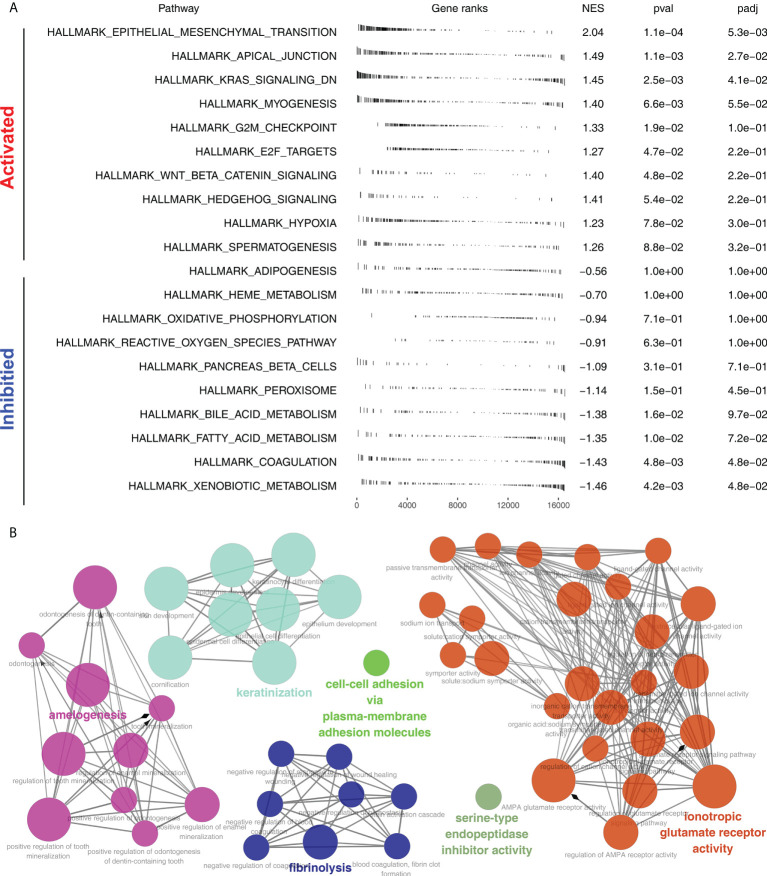
Biological enrichment analysis. **(A)** GSEA analysis of Cluster2 based on the Hallmark gene set; **(B)** ClueGO analysis of input genes.

**Figure 6:**
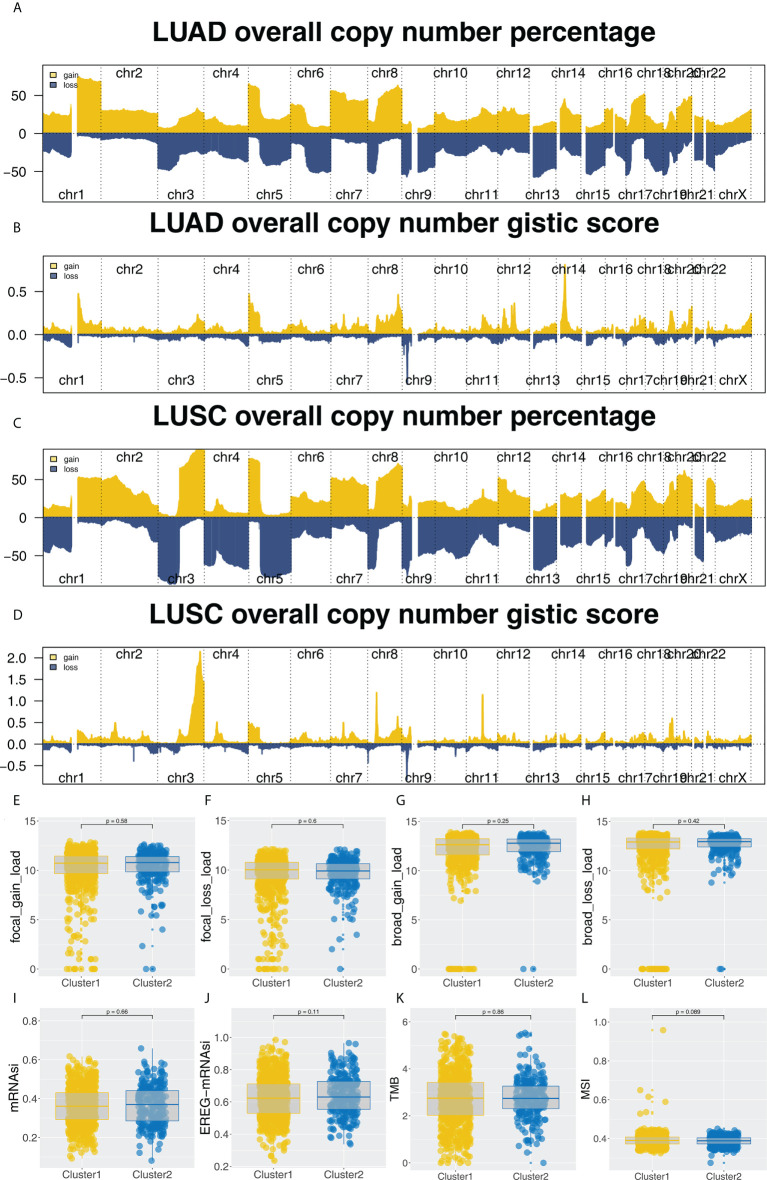
Genomic analysis. **(A-D)** The copy number percentage and gistic score of TCGA-NSCLC in Cluster1 and Cluster2; **(E-H)** The CNV burden difference in focal gain load, focal loss load, broad gain load and broad loss load level; **(I-L)** The difference of TMB, MSI, mRNAsi and EREG-mRNAsi in Cluster1 and Cluster2 patients.

Furthermore, the potential biological differences between the Cluster1 and Cluster2 patients were also explored. The result of the GSEA analysis showed that pathways of epithelial-mesenchymal transition (EMT), apical junction, KRAS signaling, myogenesis, G2M checkpoint, E2F targets, WNT/β-catenin signaling, hedgehog signaling, hypoxia were activated in Cluster2 patients (**Figure 5A**). Result of clueGO analysis indicated that the Cluster2 patients had a higher activity of amelogenesis, keratinization, fibrinolysis, serine-type endopeptidase inhibitor activity and iontropic glutamate receptor activity (**Figure 5B**). Kyoto Encyclopedia of Genes and Genomes (KEGG) analysis showed that in the Cluster2, the terms of neuroactive ligand receptor interaction, pathways in cancer, axon guidance, focal adhesion, ECM receptor interaction were enriched in (**Figure S3A**). Gene ontology (GO) analysis indicated that in the Cluster2, the terms of sensory organ development, morphogenesis of an epithelium, skeletal system development, presynapse, axon development, embryonic organ development were enriched in (**Figure S3B**). We also investigated the genomic difference between Cluster1 and Cluster2 patients. TCGA-NSCLC patients’ copy numbers profiles were investigated, including gain/loss percentages and gistic scores (**Figures 6A–D**). Nonetheless, no remarkable statistical difference was noticed in CNV burden between Cluster1 and Cluster2 patients (**Figures 6E–H**, focal gain load level, focal loss load level, broad gain load level, broad loss load level). Tumor stemness analysis showed that the patients in Cluster1 and Cluster2 might have similar tumor stemness characteristics (**Figures 6I, J**). Neither the TMB nor MSI scores were significantly different (**Figures 6K, L**).

### Female patients are more sensitive to immunotherapy

**Figure 7:**
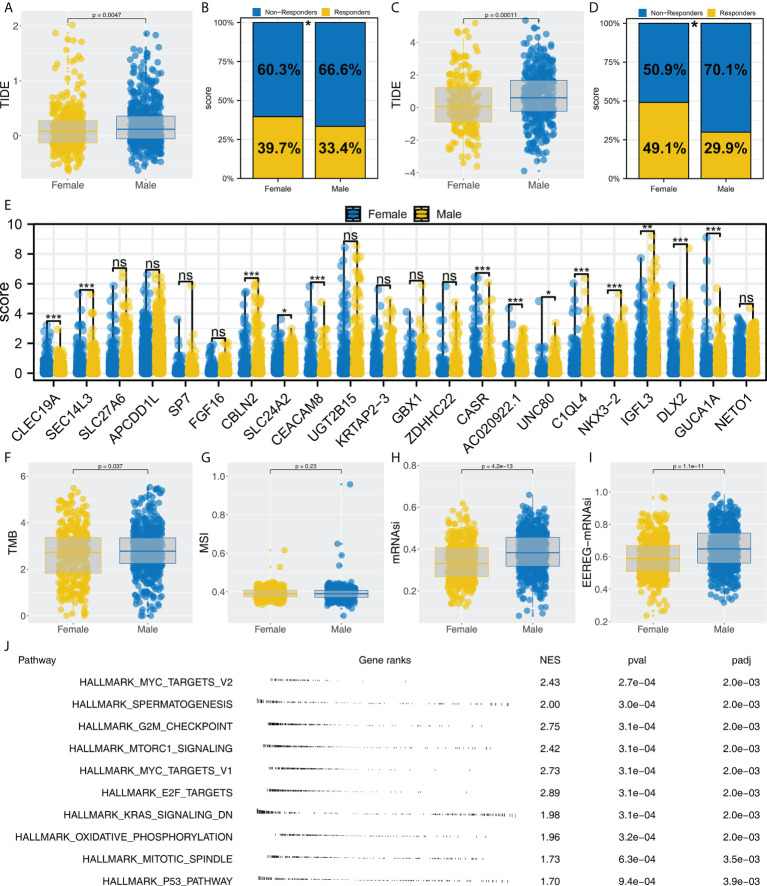
Female patients are more sensitive to immunotherapy. **(A, B)** Female patients had a lower TIDE score and higher proportion of immunotherapy responders in the TCGA cohort, * = P < 0.05; **(C, D)** Female patients had a lower TIDE score and a higher proportion of immunotherapy responders in the GEO cohort, * = P < 0.05; **(E)** The expression level of characteristic genes in male and female patients, ns = P > 0.05, * = P < 0.05, *** = P < 0.001,; **(F–I)** The difference of TMB, MSI, mRNAsi and EREG-mRNAsi in female and male patients; **(J)** Biological enrichment was performed to explore the underlying difference in female and male patients.

We noticed that Cluster1 patients had a higher percentage of female patients in both TCGA and GEO cohorts. Therefore, we speculated whether there is a potential difference in immunotherapy between male and female NSCLC patients. Our findings from the TCGA cohort indicated that patients who respond to immunotherapy are more likely to be female and have a lower TIDE score (**Figures 7A, B**, 39.7% *vs*. 33.4%). Also, the same conclusion was found in the combined GEO cohort (**Figures 7C, D**, 49.1% *vs*. 29.9%). Moreover, we found several immunotherapy characteristic genes were differentially expressed in female and male patients, including CBLN2, SLC24A2, CEACAM8, CASR, AC020922.1, UNC80, C1QL4, NKX3-2, IGFL3, DLX2 and GUCA1A (**Figure 7E**). Interestingly, a significantly increased TMB, mRNAsi and EREG-mRNAsi were noticed in male patients, but not MSI (**Figures 7F–I**). GSEA analysis showed that compared with female patients, pathways of MYC target, G2M checkpoints, mTORC1 signaling, MYC target, E2F target, KRAS signaling, oxidative phosphorylation, mitotic spindle and P53 pathway were activated (**Figure 7J**).

### Monocytes have a potential role in affecting NSCLC immunotherapy

**Figure 8:**
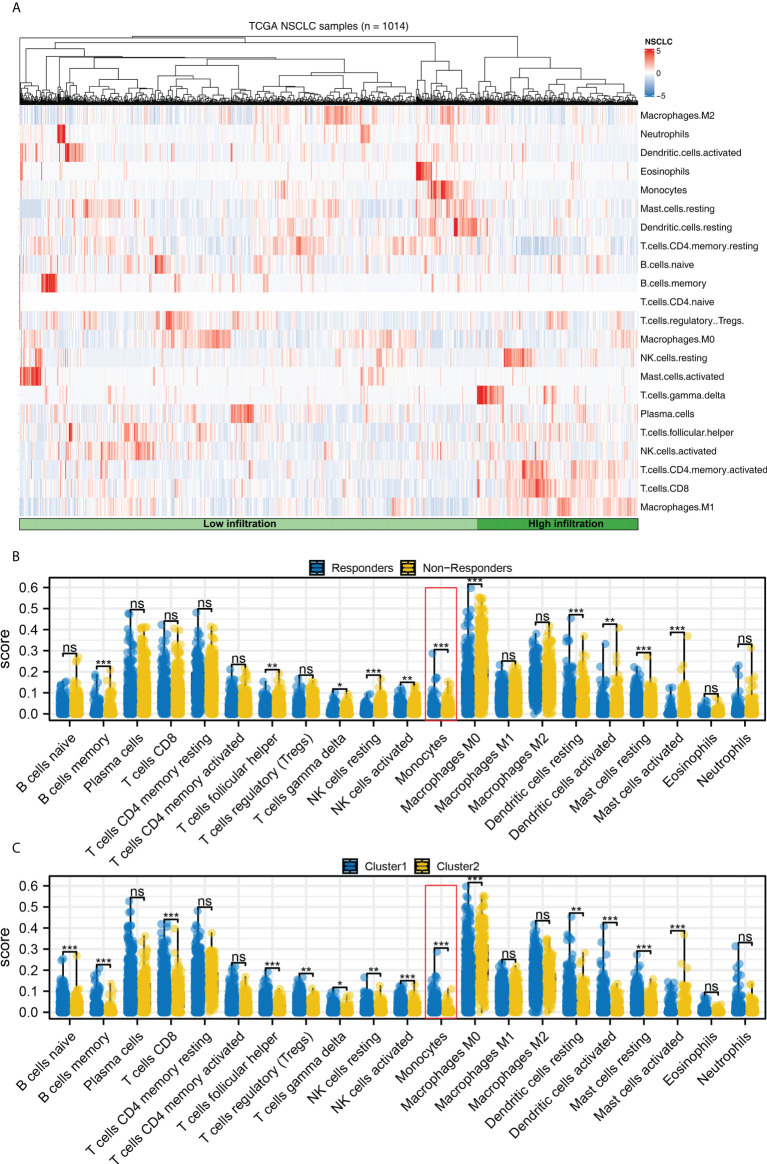
Immune infiltration. **(A)** The CIBERSORT algorithm was used to quantify the immune cell infiltration; **(B)** The immune cell infiltration level in immunotherapy responders and non-responders, ns = P > 0.05, * = P < 0.05, *** = P < 0.001; **(C)** The immune cell infiltration level in Cluster1 and Cluster2 patients, ns = P > 0.05, * = P < 0.05, ** = P < 0.01, *** = P < 0.001.

**Figure 9:**
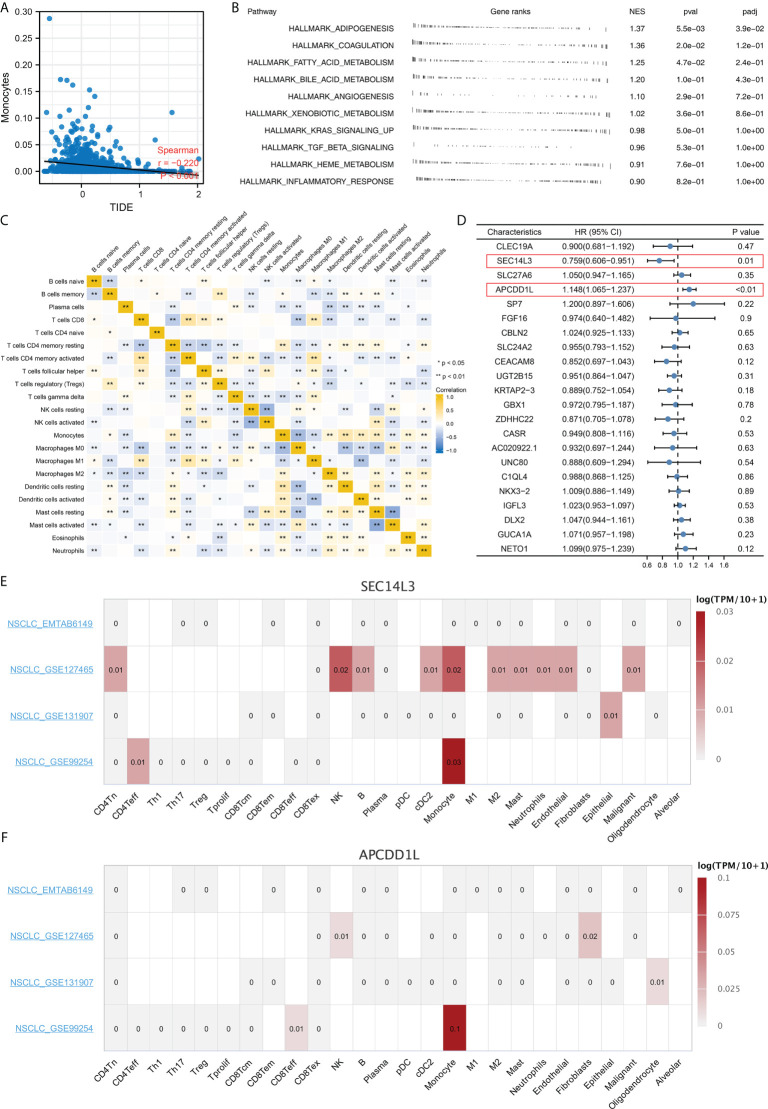
Monocytes have a potential role in affecting NSCLC immunotherapy. **(A)** Monocytes was negatively correlated with TIDE score; **(B)** Biological enrichment analysis of monocytes; **(C)** Correlation of quantified immune cells; **(D)** Among all the characteristic genes, SEC14L3 and APCDD1L were identified as prognosis-related based on the univariate Cox regression analysis; **(E, F)** Single cell analysis of SEC14L3 and APCDD1L based on the TISCH website. *P < 0.05; **P < 0.01.

Complex immune microenvironment can affect the immunotherapy of NSCLC patients. Thus, we quantified the immune microenvironment (22 immune cells) using CIBERSORT algorithm (**Figure 8A**). We found that the activated dendritic cells, M0 macrophages, memory B cells, follicular helper T cells, resting NK cells, monocytes, resting dendritic cells, resting mast cells, γδ T cells, activated NK cells, activated mast cells had a different infiltration pattern in immunotherapy responders and non-responders patients (**Figure 8B**). Additionally, the naive and memory B cells, CD8 T cells, activated mast cells, resting NK cells, regulatory T cells, γδ T cells, activated NK cells, resting dendritic cells, monocytes, activated dendritic cells, resting mast cells, follicular helper T cells had a different infiltration pattern in Cluster1 and Cluster2 patients (**Figure 8C**). A negative correlation was found between monocytes and the calculated TIDE score (**Figure 9A**, correlation = -0.220, P < 0.001). For the patients with high monocytes infiltration, pathways of adipogenesis, coagulation, fatty acid metabolism, bile acid metabolism, angiogenesis, xenobiotic metabolism, KRAS signaling, TGF-β signaling, heme metabolism and inflammatory response were activated (**Figure 9B**). The correlation between quantified immune cells based on the CIBERSORT algorithm was shown in **Figure 9C**. Among all the characteristic genes, SEC14L3 and APCDD1L were identified as prognosis-related based on the univariate Cox regression analysis (**Figure 9D**). SEC14L3 and APCDD1L are primarily expressed in monocytes, based on single-cell analysis (**Figures 9E, F**). These results revealed that monocytes have a potential role in affecting NSCLC immunotherapy and identified SEC14L3 and APCDD1L as the underlying targets.

## Discussion

In patients with NSCLC, although early diagnosis and surgical treatment have been shown to greatly improve cure rates, the prognosis remains poor (14). Among NSCLC treatments, immunotherapy is considered a promising strategy (15). Recent studies have shown that PD-1/L1 inhibitors can effectively increase survival over chemotherapy (16). However, it is hard to accurately predict how NSCLC will respond to immunotherapy (17). In addition, most patients do not respond to immunotherapy, deteriorate during treatment, or suffer severe immunotoxicity since the indications for immunotherapy are not understood (18). Therefore, to maximize the effectiveness of immunotherapy, it is necessary to identify biomarkers that are associated with immunotherapy response.

In our study, characteristic genes were identified through two machine learning algorithm, LASSO logistic and SVM-RFE regression. SVM-RFE regression can determines the best variable by deleting the SVM feature vector. Meanwhile, the A Lasso logistic regression determines variables by searching for the smallest classification error λ. Nowadays, the massive data generated by next-generation sequencing not only brings convenience for research, but also brings redundancy of data. Through dimensionality reduction, machine learning algorithm can effectively identify the characteristic variables of specific groups. In the clinical practice, detecting the expression level of identified characteristic genes through gene chip can indicate the immunotherapy response of patients, further guiding therapy option.

Based on the results of GSEA, the difference between Cluster2 and Cluster1 groups was associated with EMT, apical junction, KRAS signaling, Wnt/β-catenin signaling, Hedgehog signaling and E2F target. According to a previous study, EMT-related genes are highly accurate predictors of immune checkpoint inhibitor response in advanced NSCLC patients (19). Another study revealed that clinical benefit has been demonstrated in previously treated KRAS G12C-mutant NSCLC patients who received immunotherapy of sotolacide and adagracil (20). Further, based on the Hedgehog signaling and Wnt/β-catenin, various immunotherapies have been developed for NSCLC. Yoshiko et al. discovered that WNT/β-catenin signaling inhibitor and PD-1 blocker combination therapy improved antitumor immunity in NCSLS and suggested a mechanism-oriented combination therapy (21). For Hedgehog signaling, researchers found that targeting Hedgehog signaling could offer therapeutic benefits to patients with NSCLC (22). According to the GSEA, the Cluster1 group was associated with the xenobiotic metabolism, fatty acid metabolism, bile acid metabolism, peroxisome and reactive oxygen species pathway. Currently, the reactive oxygen species pathway is a potential target for immunotherapy of NSCLC. Additionally, it has been shown that the NRF2, which is involved in the reactive oxygen species pathway, can inhibit the immune response of NSCLC patients and promote the immune escape of tumor cells (22). In NSCLC patients, fatty acid oxidation has broad therapeutic potential. It is believed that fatty acid oxidation increases mitochondrial mass, which in turn suppresses T-cell immunity, promoting NSCLC progression (23). Our result showed that the enriched pathway above might be responsible for the prognosis and immunotherapy response difference between the patients in Cluster1 and Cluster2.

Further research discovered that female and male distributions were significantly different between Cluster1 and Cluster2. We also discovered a lower immune response rate in male NSCLC patients, while a higher immune response rate is observed in female NSCLC patients. Recent research has demonstrated that men and women respond differently to NSCLC and immunotherapy due to differences in the immune system (24). NSCLC cells may be exposed to a more effective immune surveillance mechanism when estrogen regulates the production of inflammatory cytokines from macrophages and neutrophils (25). Subsequently, immune infiltration analysis indicated a significant difference in monocyte distribution between Cluster1 and Cluster2. According to the univariate cox regression analysis, SEC14L3 and APCDD1L are risk factors for NSCLC survival. Single-cell transcriptomics of lung cancers reveals that SEC14L3 and APCDD1L were also enriched in monocyte. According to studies combining anti-angiogenic and targeted immunotherapy, immunotherapy is influenced by the tumor microenvironment, which is a potential target for developing novel immunotherapy drugs (26). As a key regulator in NSCLC progression, monocytes can drive an aggressive phenotype in NSCLC (27). In a clinical study, absolute monocyte counts in peripheral blood were found to be a good predictor of outcomes in NSCLC patients treated with immunotherapy (28). In this work, underlying targets like monocytes, SEC14L3 and APCDD1L were identified, which can be improved to be more personalized NSCLC immunotherapy in the future.

In all, our study comprehensively explored the underlying differences between immunotherapy responders and non-responders. We identified characteristic genes and performed molecular subtyping to screen patients with different responses to immunotherapy. Interestingly, we found that female patients were more sensitive to immunotherapy; monocytes have a potential role in affecting NSCLC immunotherapy; SEC14L3 and APCDD1L were the underlying targets affecting immunotherapy, as well as patients survival. These results can provide direction and guidance for future research focused on NSCLC immunotherapy. However, our study also exists some limitations. Firstly, in our analysis, White patients constituted the majority, indicating that race bias is unavoidable. It is important to pay more attention to large-scale sequencing data from Asia and Africa in the future. Secondly, the genomic data of NSCLC patients treated with immunotherapy is still not openly accessible. In practice, the response rate predicted by TIDE analysis does not fully reflect reality.

## Data availability statement

Publicly available datasets were analyzed in this study. This data can be found here: https://portal.gdc.cancer.gov/%20 and https://www.ncbi.nlm.nih.gov/gds/?term=.

## Author contributions

XZ, XW, and HH collected the data and performed the analysis. XZ and KD wrote the manuscript. YN, PS, and YL designed the work. All authors contributed to the article and approved the submitted version.

## Conflict of interest

The authors declare that the research was conducted in the absence of any commercial or financial relationships that could be construed as a potential conflict of interest.

## Publisher’s note

All claims expressed in this article are solely those of the authors and do not necessarily represent those of their affiliated organizations, or those of the publisher, the editors and the reviewers. Any product that may be evaluated in this article, or claim that may be made by its manufacturer, is not guaranteed or endorsed by the publisher.

## Supplementary material

Supplementary Figure 1 The flow chart of whole study.

Supplementary Figure 2 Molecular typing based on characteristic genes in the TCGA database.

Supplementary Figure 3 GO and KEGG analysis.
