# Supplementary figures and images for: Research into the biological differences and targets in lung cancer patients with diverse immunotherapy responses
Source: Front Immunol. 2022 Sep 16;13:1014333. doi: 10.3389/fimmu.2022.1014333 (PMC9523410; doi:10.3389/fimmu.2022.1014333)

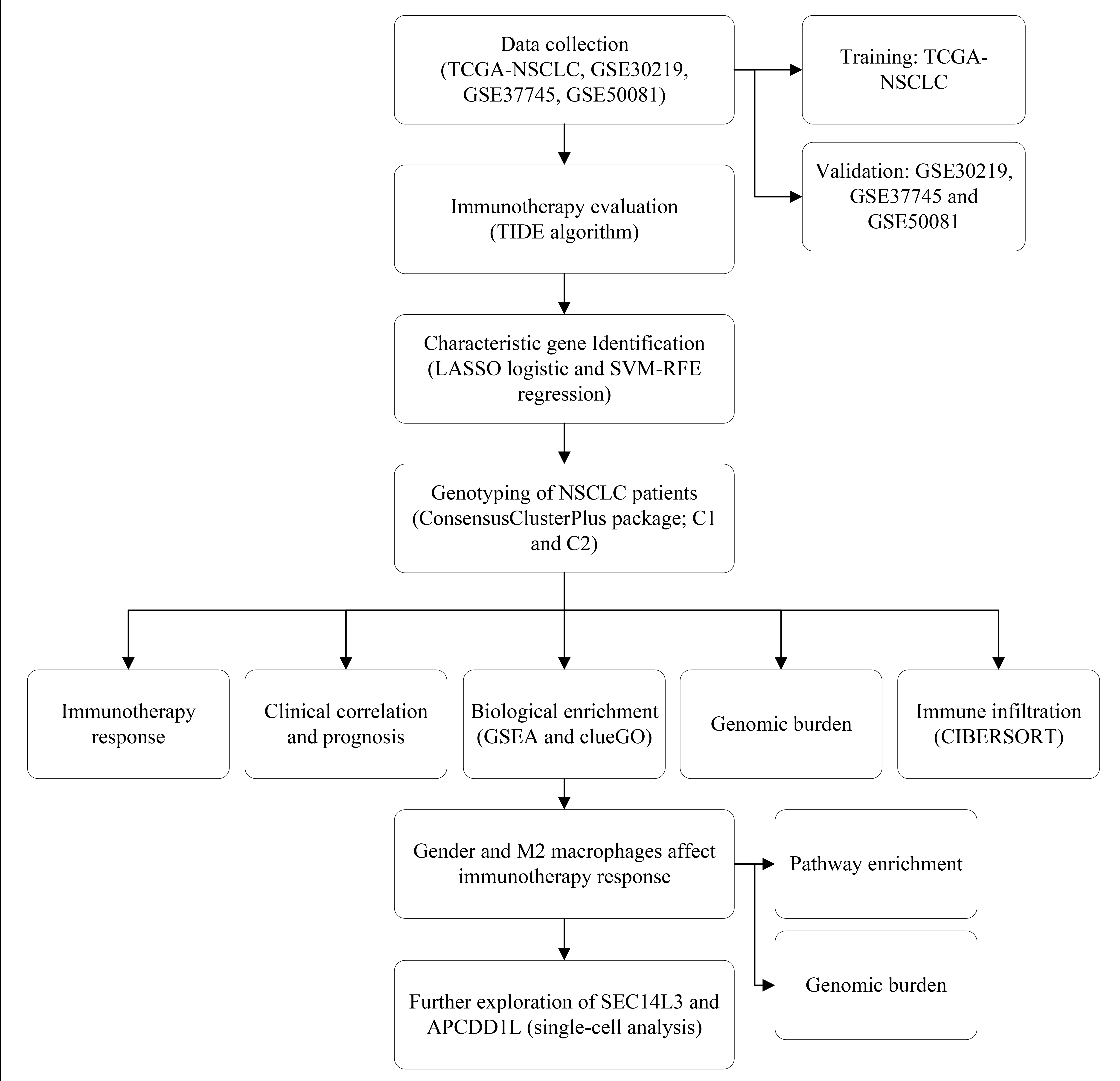

Supplement: Supplementary Figure 1 — The flow chart of whole study. [file Image_1.tif]

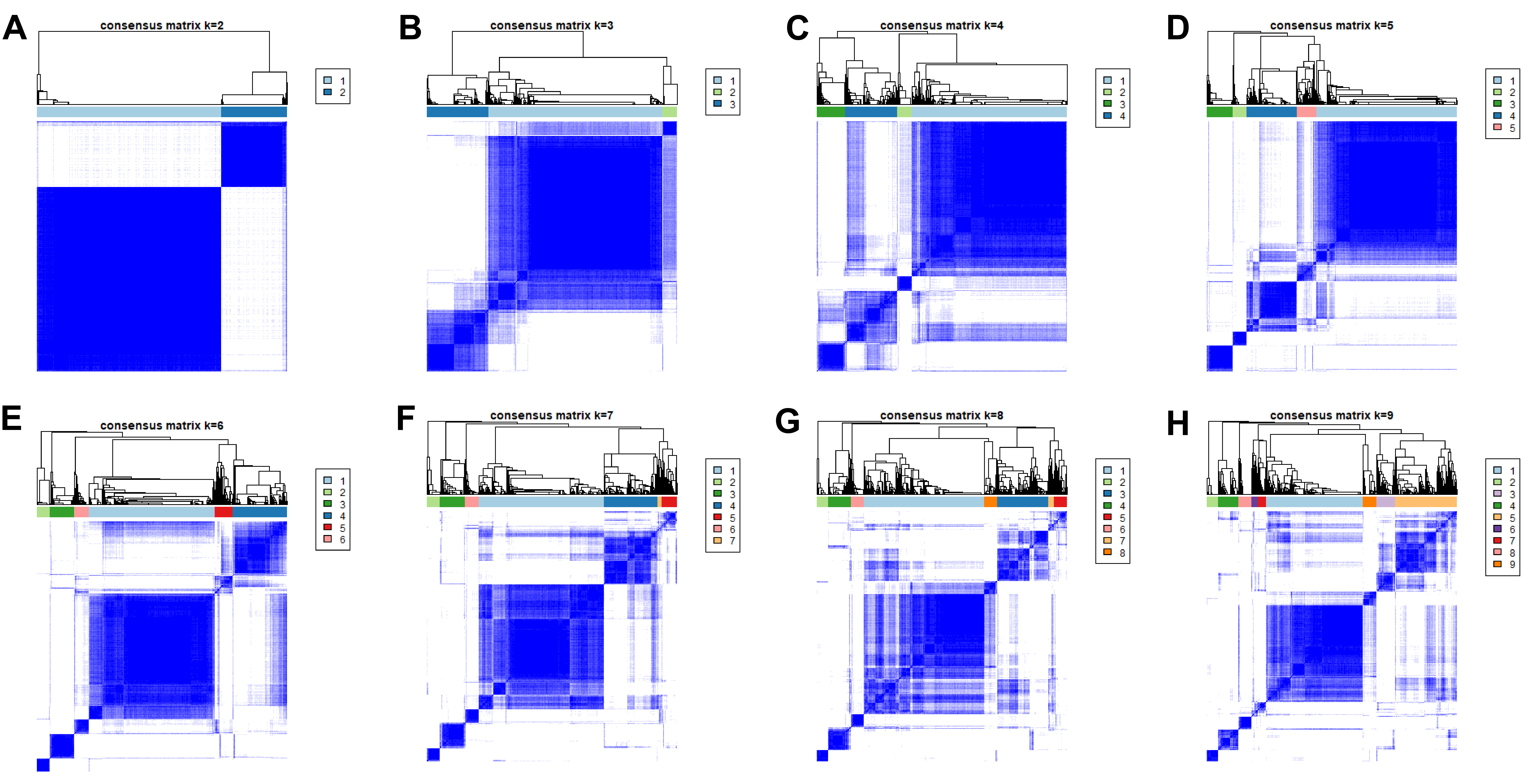

Supplement: Supplementary Figure 2 — Molecular typing based on characteristic genes in the TCGA database. [file Image_2.tif]

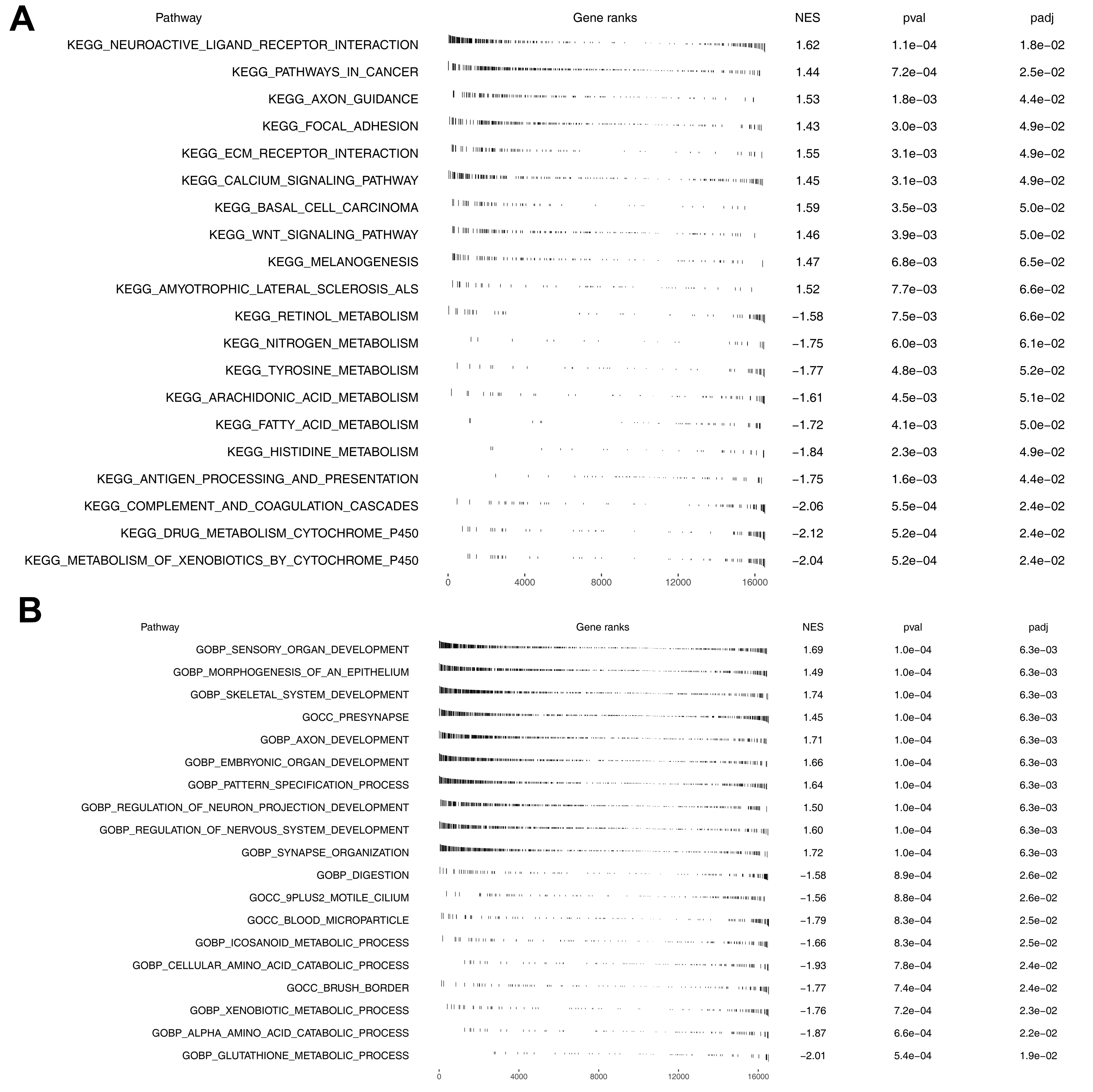

Supplement: Supplementary Figure 3 — GO and KEGG analysis. [file Image_3.tif]
